# Human papillomavirus molecular prevalence in south China and the impact on vaginal microbiome of unvaccinated women

**DOI:** 10.1128/msystems.00738-24

**Published:** 2024-08-09

**Authors:** Tingting Wang, Weili Li, Mingya Cai, Shushen Ji, Yufang Wang, Nan Huang, Yancheng Jiang, Zhishan Zhang

**Affiliations:** 1 School of Health, Quanzhou Medical College, Quanzhou, China; 2 Zhangjiang Center for Translational Medicine, Shanghai Biotecan Pharmaceuticals Co., Ltd., Shanghai, China; 3 Department of Clinical Laboratory, Quanzhou First Hospital Affiliated to Fujian Medical University, Quanzhou, China; 4 Department of Clinical Laboratory, Jinjiang Hospital, Jinjiang, China; University of California San Diego, La Jolla, California, USA

**Keywords:** human papillomavirus, prevalence, vaginal microbiome, cervical intraepithelial neoplasia

## Abstract

The vaginal microbiome (VM) is associated with human papillomavirus (HPV) infection and progression, but a thorough understanding of the relation between HPV infection, and VM needs to be elucidated. From August to December 2022, women who underwent routine gynecological examinations were screened for HPV infection. The distribution of HPV variants and clinical characteristics were collected. Then, a total of 185 participants were enrolled and divided into HPV-negative (HC), high-risk HPV (H), low-risk HPV (L), multiple high-risk HPV (HH), and mixed high-low risk HPV (HL) groups. Samples were collected from the mid-vagina of these 185 participants and sent for 16S rDNA sequencing (V3–V4 region). Among 712 HPV-positive women, the top 3 most frequently detected genotypes were HPV52, HPV58, and HPV16. Among 185 participants in the microbiology study, the β diversity of the HC group was significantly different from HPV-positive groups (*P* < 0.001). LEfSe analysis showed that *Lactobacillus iners* was a potential biomarker for H group, while *Lactobacillus crispatus* was for L group. Regarding HPV-positive patients, the α diversity of cervical lesion patients was remarkably lower than those with normal cervix (*P* < 0.05). Differential abundance analysis showed that *Lactobacillus jensenii* significantly reduced in cervical lesion patients (*P* < 0.001). Further community state type (CST) clustering displayed that CST IV was more common than other types in HC group (*P* < 0.05), while CST I was higher than CST IV in H group (*P* < 0.05). Different HPV infections had distinct vaginal microbiome features. HPV infection might lead to the imbalance of *Lactobacillus* spp. and cause cervical lesions.

**IMPORTANCE:**

In this study, we first investigated the prevalence of different HPV genotypes in south China, which could provide more information for HPV vaccinations. Then, a total of 185 subjects were selected from HPV-negative, high-risk, low-risk, multiple hr-hr HPV infection, and mixed hr-lr HPV infection populations to explore the vaginal microbiome changes. This study displayed that HPV52, HPV58, and HPV16 were the most prevalent high-risk variants in south China. In addition, high-risk HPV infection was featured by *Lactobacillus iners*, while low-risk HPV infection was by *Lactobacillus crispatus*. Further sub-group analysis showed that *Lactobacillus jensenii* was significantly reduced in patients with cervical lesions. Finally, CST clustering showed that CST IV was the most common type in HC group, while CST I accounted the most in H group. In a word, this study for the first time systemically profiled vaginal microbiome of different HPV infections, which may add bricks to current knowledge on HPV infection and lay the foundation for novel treatment/prevention development.

## INTRODUCTION

Human papillomavirus (HPV) are ubiquitous double-stranded DNA viruses that can be transmitted sexually. Nowadays, hundreds of HPV genotypes have been reported and many of them can infect the genital tract (1). Genital HPV infections can be asymptomatic, and most women are reported to have HPV infections at some time throughout their lives. Although over 90% can be cleared spontaneously (2), there is still a small proportion of women suffering from persistent infection, which may lead to cervical neoplastic lesions (3, 4). According to a recent largest epidemiology study in China (5), the overall prevalence of HPV was more than 20% and was increasing over the years. Though HPV vaccination is an effective prevention method (6, 7), the vaccine coverage is still low in mainland China (8). In addition, HPV vaccines only protect women from targeted HPV genotypes, leaving them still at risk of other genotypes of HPV infections. Considering that concurrent multiple HPV infections are common (9, 10) and are associated with persistent infections (11, 12), the role of multiple HPV infections in cervical cancer development should not be overlooked. In addition, based on the carcinogenicities, HPV can be divided into high-risk HPV (hr-HPV) and low-risk HPV (lr-HPV). Hr-HPV consists of at least 12 genotypes, including HPV16, HPV18, HPV52, and so on. Worldwide, the prevalence of hr-HPV genotypes demonstrated a region-specific pattern. Typically, HPV18 is reported to be the most common one in Europe, while HPV16 is one of the most frequent variant in Africa, North America, and China (13, 14). Emerging evidence showed that the biological behaviors of hr-HPV and lr-HPV were different regarding viral genome integration (15, 16), and such variance could explain the distinct outcomes of the two types of HPV infection. But, beyond these aspects, other mechanisms underneath the pathogenicity differences deserve exploring.

Like the human gut and skin microbiome, abundant microorganisms reside in the vagina, playing essential roles in regulating vaginal microecological balances. To date, extensive work has been done regarding the constitution of vaginal microbiota. For instance, Ravel et al*.* (17) reported that bacterial communities in asymptomatic women can be classified into five community state types (CSTs). CST I, II, III, and V were dominated by *Lactobacillus crispatus*, *Lactobacillus gasseri*, *Lactobacillus iners*, and *Lactobacillus jensenii*, respectively, while CST IV was featured by a more diverse community. Their research also showed that CST III accounted for the most proportion of the Asian women, followed by CST I and CST IV. Rapidly accumulating research showed that the vaginal microbiota was also involved in HPV acquisition and persistence (18), and the relationships between vaginal microbiome and HPV infections are increasingly understood. A systemic research that enrolled 15 prospective cohort studies found a causal link between vaginal dysbiosis and HPV infection and persistence (19). Lee et al. (20) reported a higher diversity, a lower level of *Lactobacillus*, especially *L. iners*, in the HPV-positive group. Vargas-Robles et al. (21) further investigated the cervicovaginal microbiome of Hispanic women regarding physiological stages, HPV infection types, as well as cervical lesions. In recent years, increasing studies explored the vaginal microbiome features of hr-HPV (22, 23), lr-HPV (24), and multiple HPV infections (25). A comprehensive depiction of the vaginal microbiome of patients with different types of HPV infection will provide an expanded understanding of relationships between vaginal microbiota, HPV, and cervical cancers among the Chinese population.

This study firstly investigated the distribution of different HPV genotypes among women in Quanzhou, providing an overview of HPV infections in south China. Then, further exploration of the vaginal microbiome in patients with distinct types of HPV infection was performed using next-generation sequencing. By depicting vaginal microbial profiles of the hr-/lr-/multiple HPV infections, more in-depth knowledge on the mechanisms of HPV infection could be obtained, laying the foundation for novel therapy development to prevent HPV infection and restore cervicovaginal health.

## MATERIALS AND METHODS

### Study design and specimen collection

**FIG 1:**
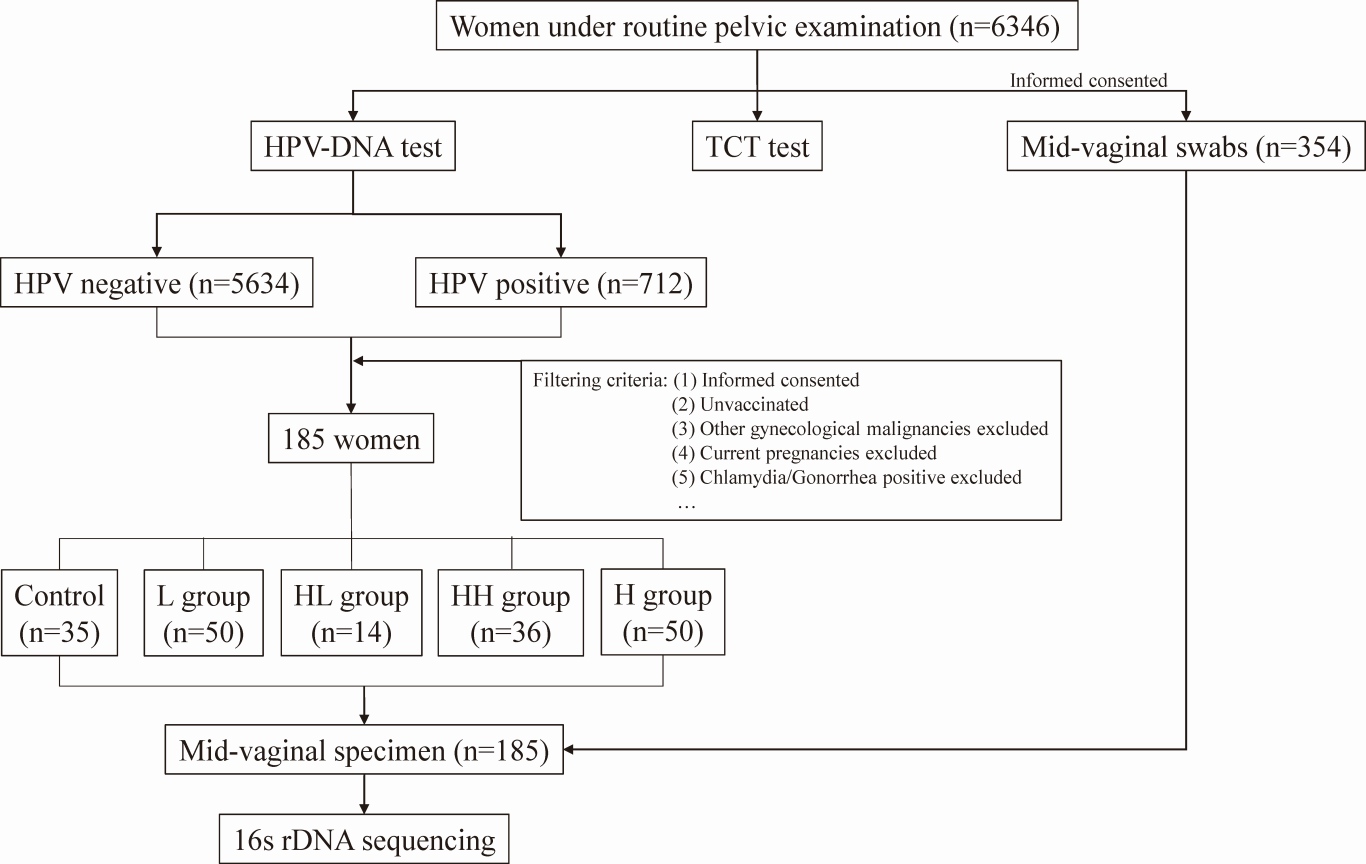
The flowchart of the study design.

From August to December 2022, a total of 6,346 women underwent routine gynecological examinations in the Department of Gynecology of the First Hospital of Quanzhou City, Fujian, China. Cervical specimens were collected with a Cytobrush and were sent for cervical Thinprep Cytology Test (TCT) (Hologic, Marlborough, MA, USA) and HPV DNA detection, respectively. HPV DNA test was performed with an HPV detection kit according to the manufacturer’s instruction (Hybribio, Chaozhou, Guangdong, China), which uses PCR + membrane hybridization technique and can qualitatively detect 21 types of HPV DNA, namely, 6, 11, 53, 16, 18, 31, 33, 58, 35, 39, 45, 51, 52, 56, 59, 66, 68, 42, 43, 44, and CP8304 (often infringes Chinese population [5]). Among these, 14 genotypes were hr-HPV, including HPV16, 18, 31, 33, 35, 39, 45, 51, 52, 56, 58, 59, 66, and 68; 6 genotypes were lr-HPV, including HPV6, 11, 42, 43, 44, and CP8304, while 1 genotype, that is, HPV53, was intermediate-risk HPV. Histopathological examinations were performed on patients according to China Cervical Cancer Diagnoses and Treatment Guidelines (26). Patients with cervical lesions were further classified into low-grade squamous intraepithelial lesions (LSIL), high-grade squamous intraepithelial lesions (HSIL), and squamous-cell carcinoma (SCC) based on the WHO Classification of Female Genital Tumors (27), which indicated cervical intraepithelial neoplasia grade 1 (CIN1) to be LSIL, while CIN2 and CIN3 to be HSIL. Then, a third specimen was collected from the mid-vagina of subjects who gave informed consent (*n* = 354) and was immediately stored at −80°C. From these individuals, a total of 185 age-matched subjects were included in the vaginal microbiome study, containing 35 subjects with HPV-negative (HC), 50 with single hr-HPV infections (H), 50 with single lr-HPV infections (L), 36 with multiple hr-hr HPV infections (HH), and 14 with hr-lr HPV infections (HL). The respective third specimens of these participants were shipped with sufficient dry ice and were subjected to 16S rDNA sequencing. The exclusion criteria were as follows: (i) HPV vaccinated; (ii) other gynecological cancer types except cervical cancer; (iii) current pregnancy; (iv) chlamydia/gonorrhea/HIV infection; (v) sexual intercourse or vaginal lavage in 3 days before sample collection; and (vi) antibiotics or vaginal antimicrobials usage in 2 weeks before sample collection. The clinical characteristics of these participants were recorded, including age, body mass index (BMI), marital status, gravity times, parity times and vaginal discharge examination results. The flowchart of the study design can be found in Fig. 1. The study protocol was approved by the ethics committee of the First Hospital of Quanzhou City (#2021010), and the study was conducted following the principles of the Declaration of Helsinki. All participants in the microbiological study provided written informed consent before sample collection. The STORMS checklist can be found at https://doi.org/10.6084/m9.figshare.25440403.

### Bacterial DNA extraction and 16S rDNA sequencing

First, according to the manufacturer’s instructions, the total bacterial genomic DNA was extracted with the PowerMax DNA isolation kit (MoBio Laboratories, Carlsbad, CA, USA) and stored at −20°C. A NanoDrop ND-1000 spectrophotometer (Thermo Fisher Scientific, Waltham, MA, USA) and agarose gel electrophoresis were performed to test the quantity and quality of extracted DNA, respectively. Then, the V3–V4 region of the bacterial 16S rDNA gene was amplified using polymerase chain reaction (PCR) with the forward primers 341F (5′-CCTAYGGGRBGCASCAG-3′) and the reverse primer 806R (5′-GGACTACHVGGGTWTCTAAT-3′). The PCR was performed in a reaction system that contained 25 µL of Phusion High-Fidelity PCR Master Mix, 3 µL (10 μM) of forward primer, 3 µL (10 μM) of reverse primer, 10 µL of DNA template, 3 µL of DMSO, and 6 µL of ddH_2_O. The thermal cycling conditions were set as initial denaturation at 98°C for 30 s, followed by 25 cycles consisting of denaturation at 98°C for 15 s, annealing at 58°C for 15 s, and extension at 72°C for 15 s, with a final extension of 1 min at 72°C. Next, PCR amplicons were purified by Agencourt AMPure XP Beads (Beckman Coulter, Indianapolis, IN) and quantified with the PicoGreen dsDNA Assay Kit (Invitrogen, Carlsbad, CA, USA), followed by pooling of the amplicons in equimolar quantities. After that, pair-end 2 × 150 bp sequencing was performed on the Illumina NovaSeq 6000 platform at Shanghai Biotecan Co., Ltd (Shanghai, China).

### Bioinformatic analysis

Raw sequencing reads that matched exactly to the barcodes were assigned to respective samples. Then, quality filtration of raw reads was performed following the below criteria: sequences that (i) had a length under 150 bp; (ii) had average Phred scores under 20; (iii) contained ambiguous bases; and (iv) contained mononucleotide repeats over 8 bp were removed. Next, paired-end reads were assembled using Vsearch v2.4.4, and unique sequences were assigned to operational taxonomic units (OTUs) with 97% similarity by mothur (v1.39.5). The taxonomy of these OTUs was further generated by searching against the Greengenes2 database. An OTU table recording the abundance of each OTU in a single sample was further generated. Before downstream analysis, the rarefy function from the vegan R package was used to perform rarefaction on the OTU table, normalizing it to the minimum sequence count across all samples. Moreover, to minimize the differences in sequencing depth across samples, OTUs that contained lower than 0.001% of total sequences were discarded and an averaged rarefied OTU table was generated.

The Quantitative Insights into Microbial Ecology (QIIME2, v2023.2.0) and R packages (v3.2.0) were applied to analyze sequence data. In QIIME2, the OTU table was used to calculate the α diversity indices, including the Chao1, abundance-based coverage estimator (ACE), Shannon, and Simpson indexes. Besides, β diversity analysis was also performed to explore the bacterial community’s structural differences across samples via Unifrac distance and visualized with the principal component analysis (PCA), principal coordinate analysis (PCoA), and non-metric multi-dimensional scaling (NMDS). Taxa that statistically differed in relative abundance between two groups were identified using Kruskal–Wallis test from the R stats package, and the linear discriminant analysis (LDA) effect size analysis (LEfSe) was performed with the cutoff value of the absolute LDA score (log10) being >2.0 and *P* < 0.05. In addition, ANCOM-BC2 was applied for differential abundance analysis. Finally, to predict the metagenome functions of microbials, PICRUSt2 (https://github.com/picrust/picrust2/) was applied and the functional pathways were enriched by Kyoto Encyclopedia of Genes and Genomes (KEGG) database, in addition to the human gut metabolic modules (GMMs) database and gut-brain modules (GBMs).

### Statistical analysis

For clinical characteristics, normally distributed continuous data were presented as mean ± SD, and category variables were presented as numbers and percentages. Non-parametric Dunn’s tests with Kruskal–Wallis tests or Mann–Whitney *U* test were applied in comparisons between groups. For the microbial data, the Wilcoxon rank sum test, Tukey test, and permutational multivariate analysis of variance (PERMANOVA) were used to test the differences between groups. Spearman’s rank correlation analysis was performed on featured genera and biofunctions via R (v3.2.0). All *P* values were two-sided, and *P* < 0.05 was considered statistically significant.

## RESULTS

### Epidemiology of HPV variants

**FIG 2:**
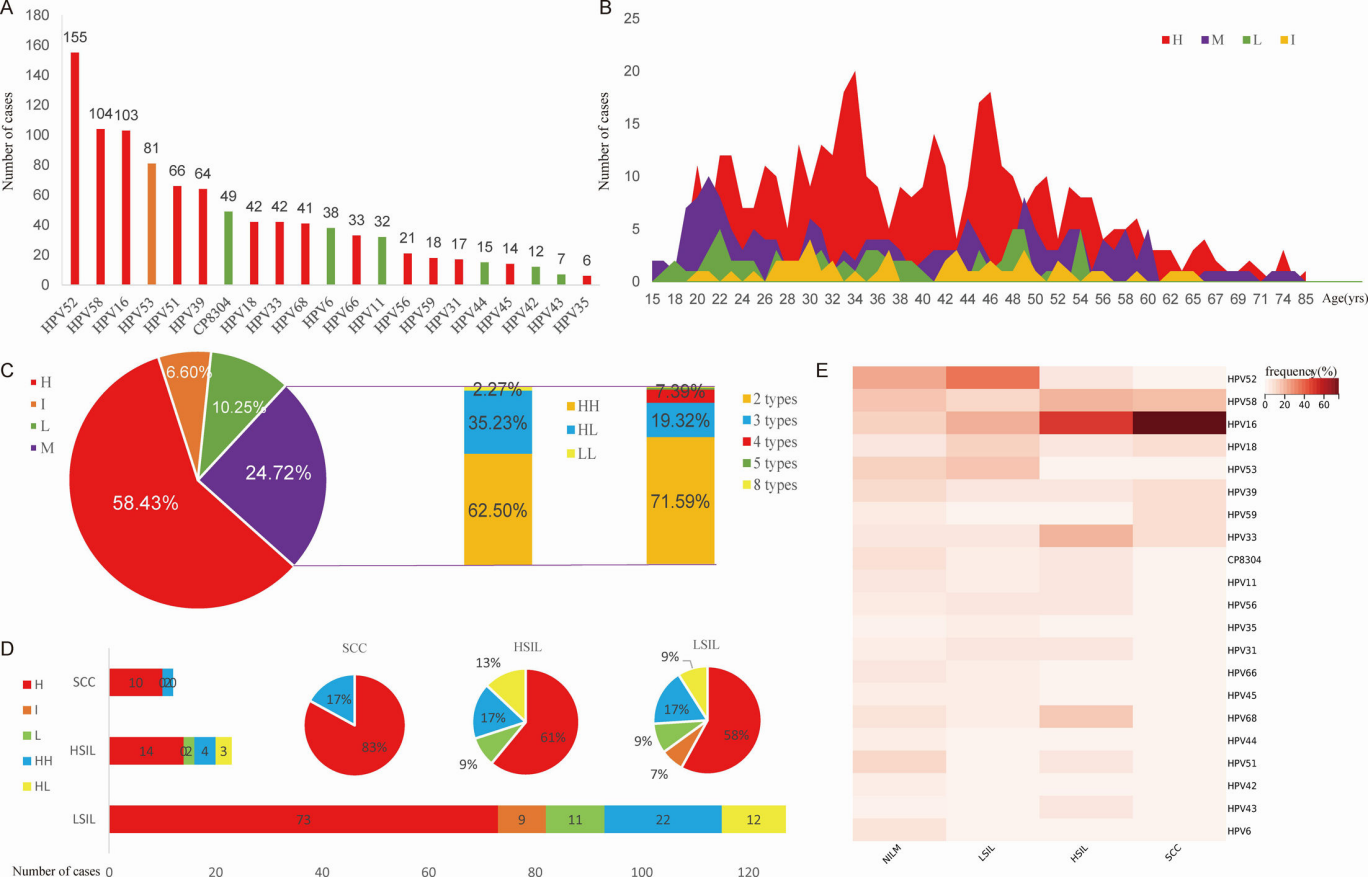
Epidemiology of HPV infections. (A) Distribution of HPV genotypes based on the present frequencies. (B) Distribution of HPV infections according to age. (C) Proportion of HPV infection types among 712 HPV-positive patients. (D) The number (bar plot) and the proportion of cases (pie graph) of HPV infection types among 162 patients with cervical lesions. (E) Frequency of HPV genotypes among patients with or without cervical lesions. H, single hr-HPV infection; HH, high-risk and high-risk multiple HPV infections; HL, high-risk and low-risk multiple HPV infections; I, single intermediate-risk HPV infection; L, single lr-HPV infection; LL, low-risk and low-risk multiple HPV infections; M, multiple HPV infections.

A total of 712 women with an average age of 39.34 ± 13.3 years old were reported to have HPV infections, and 533 of them had available cytological test results. The most frequently detected genotypes were HPV52 (155 cases), followed by HPV58 (104 cases), and HPV16 (103 cases) (Fig. 2A). Two peaks in frequencies of hr-HPV infections were observed among patients between 30 and 50 years old (Fig. 2B). The HPV distribution across age groups was displayed in Table 1. Among all patients, 416 (58.43%) had single hr-HPV infections, 47 (6.60%) had single intermediate-risk HPV infections, 73 (10.25%) had single lr-HPV infections, and 176 (24.72%) had multiple HPV infections. Among the multiple infections, 110 (62.50%) were multiple hr-hr HPV infection, 62 (35.23%) were mixed hr-lr HPV infections, while only 4 (2.27%) were multiple lr-lr HPV infections. Besides, most multiple HPV infection patients were concurrent of 2 HPV genotypes (71.59%), followed by 3 HPV genotypes (19.32%) (Fig. 2C). Among 533 patients with accessible cytological results, 162 (30.39%) had cervical lesions (including SCC, HSIL, and LSIL). The number and the proportion of different HPV infection types in cervical lesions were shown in Fig. 2D. However, there was no significant difference in cervical lesion types among HPV infection types (*P* > 0.05) (Table 2). Further analysis of the frequencies of HPV genotypes in patients with or without cervical lesions showed that HPV16 accounts for most of SCC (75%) and HSIL (48%) patients (Fig. 2E).

**TABLE 1:** Distribution of HPV infection types and age groups^*a*^

Age (years)	Single hr-HPV (*n* = 417) (%)	Single inter-risk HPV (*n* = 46) (%)	Single lr-HPV (*n* = 74) (%)	Multiple hr-hr HPV (*n* = 110) (%)	Mixed hr-lr HPV (*n* = 61) (%)	Multiple lr-lr HPV (*n* = 4) (%)	*P*-value
<25	52 (12.47)	3 (6.52)	17 (22.97)	24 (21.82)	20 (32.79)	2 (50)	0.195
25–34	118 (28.30)	15 (32.61)	16 (21.62)	22 (20)	9 (14.75)	0	
35–44	88 (21.10)	10 (21.74)	16 (21.62)	18 (16.36)	13 (21.31)	0	
45–54	103 (24.70)	12 (26.09)	23 (31.08)	23 (20.91)	13 (21.31)	1 (25)	
>54	56 (13.43)	6 (13.04)	2 (2.70)	23 (20.91)	6 (9.84)	1 (25)	

^*a*^ Data are displayed as *n* (%). *P* was calculated with Kruskal–Wallis test.

**TABLE 2:** Distribution of cytology types among different HPV infection types^*a*^

Cytology type	Single hr-HPV (*n* = 327) (%)	Single inter-risk HPV (*n* = 35) (%)	Single lr-HPV (*n* = 54) (%)	Multiple hr-hr HPV (*n* = 76) (%)	Mixed hr-lr HPV (*n* = 38) (%)	Multiple lr-lr HPV (*n* = 3) (%)	*P*-value
NILM^*b*^	230 (71.43)	26 (74.29)	41 (75.93)	48 (63.16)	23 (62.16)	3 (100)	0.315
LSIL	73 (22.67)	9 (25.71)	11 (20.38)	22 (28.95)	12 (32.43)	0	
HSIL	14 (2.80)	0	2 (3.70)	4 (5.26)	3 (5.41)	0	
SCC	10 (3.11)	0	0	2 (2.63)	0	0	

^*a*^ Data are displayed as *n* (%); the percentage was calculated as number of cytology type/number of a certain HPV infection type. *P* was calculated with Kruskal–Wallis test.

^*b*^ NILM, no intraepithelial lesion or malignancy.

### Clinical characteristics of participants in vaginal microbiome study

In the cohort of vaginal microbiome study, 185 subjects, including 50 hr-HPV (H group), 36 multiple hr-hr infections (HH group), 14 mixed hr-lr infections (HL group), 50 lr-HPV (L group), and 35 HPV-negative (HC group), were enrolled to investigate the features of vaginal microbiota. The average ages of the five groups were comparable, with 32.84 ± 1.63, 32.36 ± 6.05, 35.36 ± 6.44, 34.7 ± 6.17, and 31.49 ± 3.07, respectively. Besides, the five groups did not differ in terms of BMI, cigarette smoking, and contraception uses. Cervical lesions were found in 34% of HPV-positive patients and 6% of HPV-negative individuals. The details of demographic features of the study population can be found in Table 3.

**TABLE 3:** Clinical characteristics of 185 participants in vaginal microbiology study^*a*^

	H group (*n* = 50)	HH group (*n* = 36)	HL group (*n* = 14)	L group (*n* = 50)	HC group (*n* = 35)
Age (years, mean ± SD)	32.84 ± 1.63	32.36 ± 6.05	35.36 ± 6.44	34.7 ± 6.17	31.49 ± 3.07
BMI (kg/m^2^, mean ± SD)	20.91 ± 2.89	21.55 ± 3.15	21.56 ± 3.25	21.08 ± 3.50	20.56 ± 2.64
Current smoker (*n*, %)	0	0	0	2 (4)	0
Contraception (*n*, %)					
Condom	5 (10)	4 (11)	2 (14)	6 (12)	2 (6)
Hormonal contraception	4 (8)	3 (8)	1 (7)	9 (18)	1 (2)
IUD	4 (8)	2 (6)	1 (7)	3 (6)	3 (8)
Other	20 (40)	15 (42)	7 (50)	14 (28)	21(60)
Unknown	17 (34)	12 (33)	3 (21)	18 (36)	5 (14)
Cervical lesions (*n*, %)					
NILM	34 (68)	20 (56)	8 (57)	37 (74)	33 (94)
LSIL	10 (20)	13 (36)	4 (29)	11 (22)	2 (6)
HSIL	5 (10)	2 (6)	2 (14)	2 (4)	0
SCC	1 (2)	1 (3)	0	0	0
Gravidity (*n*, %)					
0	3 (6)	9 (25)	5 (36)	8 (16)	12 (34)
1	11 (22)	4 (11)	4 (29)	5 (10)	10 (28)
2–3	17 (34)	8 (22)	2 (14)	15 (30)	9 (26)
>3	4 (8)	3 (8)	0	6 (12)	1 (3)
Unknown	15 (30)	12 (33)	3 (21)	16 (32)	3 (9)
Parity (*n*, %)					
0	5 (10)	9 (25)	6 (43)	10 (20)	15 (43)
1	12 (24)	5 (14)	4 (27)	10 (20)	10 (28)
2	14 (28)	9 (25)	1 (7)	10 (20)	6 (17)
≥3	3 (6)	1 (3)	0	4 (8)	1 (3)
Unknown	16 (32)	12 (33)	3 (21)	16 (32)	3 (9)

^*a*^ Missing data were recorded as “unknown.” IUD, intrauterine device.

### Vaginal microbiome features with different HPV infections

**FIG 3:**
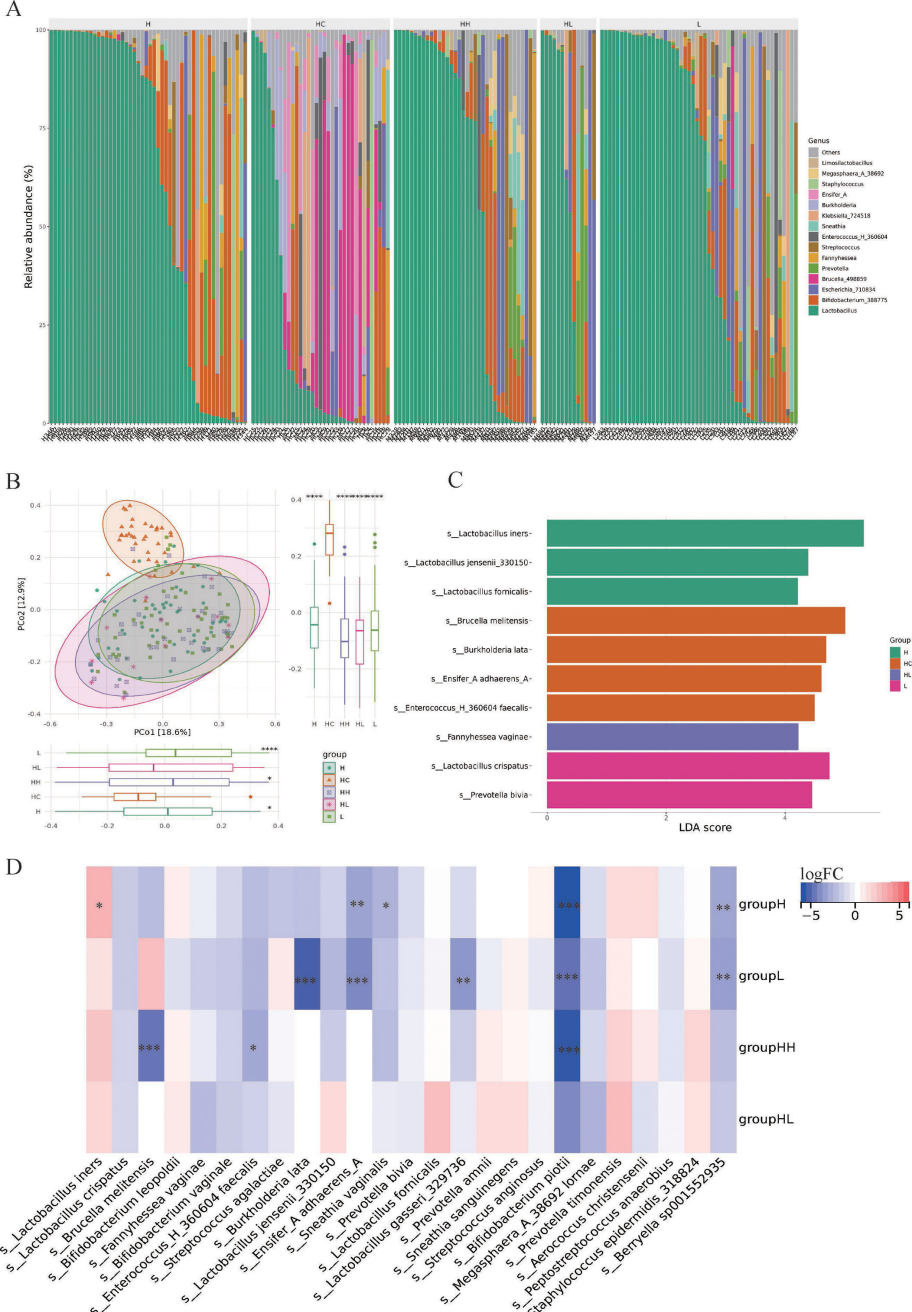
Vaginal microbiome diversity and composition among HC, H, HH, HL, and L groups. (A) Taxon at genus level across samples. (B) Differences in β-diversity of vaginal microbiome based on PCoA analysis. (C) Lefse analysis of featured microbiota of all groups. (D) Differential abundance analysis based on ANCOM-BC2. ***P < 0.001; **P < 0.01; *P < 0.05.

The microbiota composition across samples at genus level is displayed in Fig. 3A. No significant difference was found in α-diversity among the five groups (*P* > 0.05) (Fig. S1A). However, there was a significant difference in β-diversity (*P* < 0.001), with principal co-ordinates 1 and 2 explaining 18.6% and 12.9% of the variance, respectively (Fig. 3B). Typically, the HC group differed remarkably from the H group (*P* < 0.05), L group (*P* < 0.0001), and HH group (*P* < 0.05) in PCo1 and was significantly higher than the other groups in PCo2 (*P* < 0.0001). LEfSe analysis showed that the *L. iners*, *L. jensenii* 330150*,* and *Lactobacillus fornicalis* might be potential biomarkers for H group, while *L. crispatus* might be for L group (Fig. 3C). According to the differential abundance analysis performed by ANCOM-BC2, *L. iners* was significantly more abundant in H group than that in HC group (*P* < 0.05), while *s_Ensifer_A* (*P* < 0.01), *s_Bifidobacterium* (*P* < 0.001), and other bacteria were significantly lower in H group compared to HC group (Fig. 3D).

### Vaginal microbiome features in women with cervical lesions

**FIG 4:**
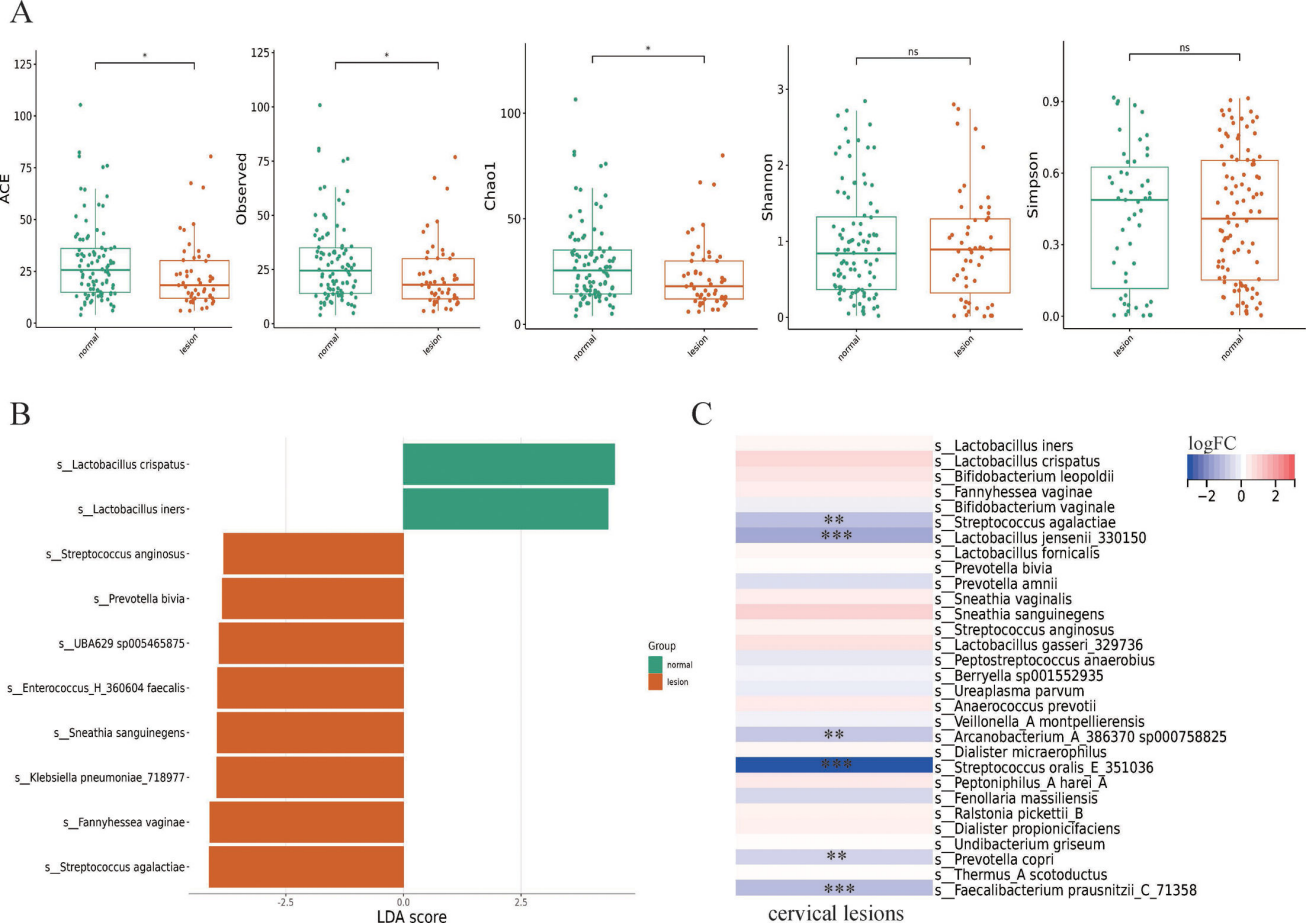
Vaginal microbiome features of patients with cervical lesions. (A) α diversity indexes of two groups. (B) Bar plot of LDA score of featured microbial (threshold LDA score > 2) based on LEfSe analysis. (C) Differential abundance analysis based on ANCOM-BC2. ***P < 0.001; **P < 0.01; *P < 0.05. *P* was calculated with the Wilcoxon test.

Among 150 HPV-positive participants, a total of 51 subjects with cervical lesions (LSIL, HSIL, SCC) were further compared with those with normal cervix to explore the differences in the vaginal microbiome. The α diversity indexes, including ACE, observed species and Chao1, were significantly lower in cervical lesion patients than those with normal cervix (*P* < 0.05) (Fig. 4A). But the β-diversity between the two groups was not significant (Fig. S1B through D). LEfSe analysis indicated that *L. crispatus* and *L. iners* were associated with normal cervix in HPV-positive patients (Fig. 4B), while *L. jensenii 330150* was remarkably lower in cervical lesion group according to the differential abundance analysis with ANCOMBC2 (*P* < 0.001) (Fig. 4C).

### CST clustering based on vaginal microbiota composition

**FIG 5:**
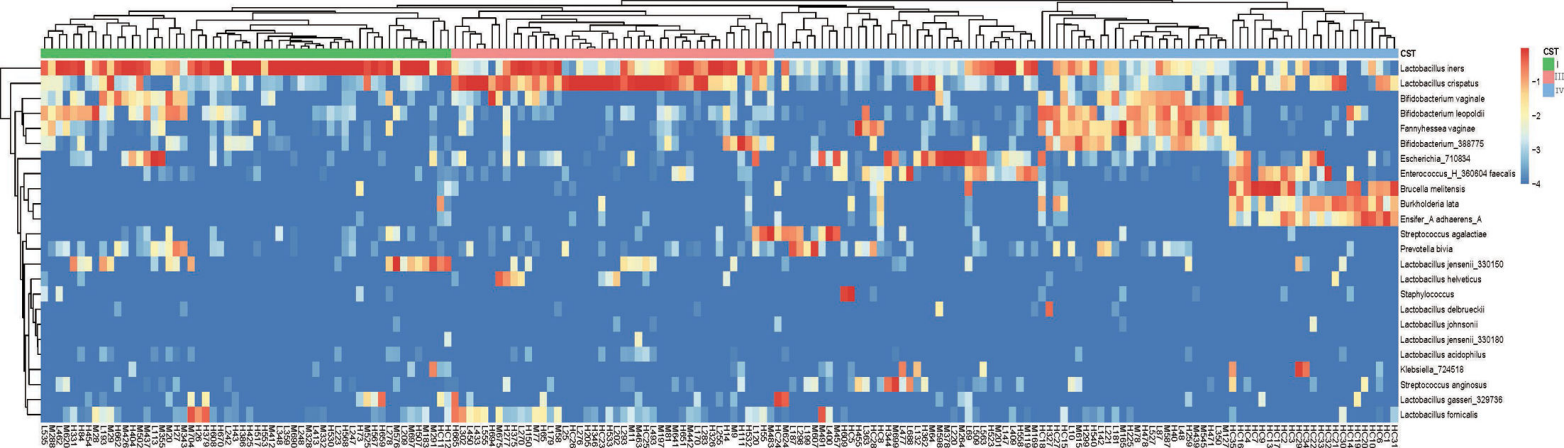
CST clustering of all samples.

According to Ravel’s (17) theory, participants in the present study were clustered into CST I (*L. crispatus*), CST III (*L. iners*), and CST IV (Diversity group). However, due to the insufficient samples, CST II and CST V were not clustered (Fig. 5). There was a significant difference in CST grouping among different HPV infection types (*P* < 0.001). Particularly, CST I accounted for a higher proportion than CST IV in H group (42% vs 28%, *P* < 0.05), while CST IV (85.7%) occurred more frequently than CST I (5.7%) and CST III (8.6%) in HC group (*P* < 0.05) (Table 4).

**TABLE 4:** HPV infection types in different CST groups^*a*^

Group	CST I (*L. crispatus*) (*n* = 56) (%)	CST III (*L. iners*) (*n* = 44) (%)	CST IV (diversity group) (*n* = 85) (%)	*P*-value
H group	21 (37.50)	15 (34.09)	14 (16.47)	<0.001
HH group	14 (25.00)	8 (18.18)	14 (16.47)	
HL group	6 (10.71)	3 (6.81)	5 (5.88)	
L group	13 (23.21)	15 (34.09)	22 (25.88)	
HC group	2 (3.57)	3 (6.81)	30 (35.29)	

^*a*^ Data are displayed as *n* (%). *P* was calculated with *χ* test.

## DISCUSSION

This cross-sectional study first investigated the prevalence of HPV variants in Quanzhou and then explored the vaginal microbiome features with different HPV infections. Consistent with other studies in Guangdong (5) and Taiwan (28), this study found that HPV52, HPV58, and HPV16 were the top three most common variants detected in south China. In addition, the distribution of HPV genotypes also displayed an age-specific feature. In this study, two peaks of hr-HPV infection were observed, with one peak appearing at 35 and the other at 45 years of age. Similarly, other studies also reported a “two-peak” phenomenon of HPV prevalence across ages. For example, Yang et al. (5) and Wang et al*.* (29) reported the two peaks of hr-HPV infection to appear at under 21 years old and around 50 years old, respectively. The underlying mechanisms for such a “two-peak” pattern remain unclear but might be the result of immunity and hormone changes over time (30). Among 533 subjects with cytological results, a total of 162 participants (30.39%) reported cervical lesions, including LSIL, HSIL, and SCC, and most of them were infected with hr-HPV or multiple hr-hr HPV infections. But there were two cases of lr-HPV infection also associated with HSIL. Furthermore, HPV16 accounted for the largest proportion of HSIL and SCC patients, which is also consistent with previous studies (1, 5, 31).

Vaginal microbiome is a dynamic “ecosystem” that maintains a healthy vaginal microenvironment. Accumulating data show that the vaginal microbiome changes under physiological and disease states (32–34). In the present study, the β diversity of HPV-positive groups, including H, L, HH, and HL groups, was remarkably different from that of HPV-negative populations, which is in agreement with other studies (24, 25). As for the bacterial taxon analysis, the abundance of *Lactobacillus,* especially *L. iners,* was higher in H group when compared to the HC group. Studies (35, 36) reported that the healthy vaginal microbiome was predominated with *Lactobacillus* and HPV-positive patients had less *Lactobacillus* spp. presence. But, not all the species of *Lactobacillus* are protective factors for cervicovaginal health. For example, *L. crispatus*, *L. jensenii*, and *L. gasseri* are noninflammatory, while *L. iners* is proinflammatory, which may promote the oncogenic potential of hr-HPV (37). Besides, a longitudinal study that explored the relationship between vaginal microbiota and HPV clearance, suggested that high abundance of *L. iners* might hinder HPV clearance in hr-HPV infections (38), highlighting the differences in bio-functions of specific *Lactobacillus* species and the importance of species identification. Although a series of studies identified potential biomarkers for HPV infections, for example, *Prevotella*, *Atopobium*, and *Dialister* were thought to be related to HPV infections (39–41), it is seldom reported about the potential biomarkers for different types of HPV infections, respectively. In this study, LEfSe analysis identified a string of featured microbiota for H, HC, HL, and L groups. To be specific, *L. crispatus* and *Prevotella bivia* might be biomarkers for L group; *Fannyhessea vaginae* for HL group; *L. iners*, *L. jensenii* 330150, and *L. fornicalis* for hr-HPV infection; and *Brucella melitensis*, *Burkholderia lata*, *Ensifer A adharens*, and *Enterococcus H 360604 faecalis* for HPV-negative individuals. Inconsistently, Wei et al*.* (22) reported an increased abundance of *Gardnerella*, *Porphyromonas*, and *Ureaplasma* in hr-HPV infections. Another study also confirmed a high abundance of *Sneathia* in lr-HPV infection patients (24). The underlying reasons for such discrepancies might probably be caused by different methodologies and pollution of background bacteria. Further sub-group analysis demonstrated that the α diversity was lower in cervical lesion group. Besides, the *L. iners* slightly but not significantly increased in cervical lesions, while *L. jensenii* remarkably reduced in patients with cervical lesions. Studies found *L. jensenii* could inhibit the viability of cervical cancer cells by regulating HPV oncogenes and cell cycle-related genes (42), and a depletion of beneficial *Lactobacillus* species may lead to a vulnerable microenvironment of female reproductive tract (43). Taken together, it can be speculated that hr-HPV infection might be responsible for *Lactobacillus* spp. imbalance, which could lead to cervical lesions.

Lactic acid, acetate, succinic acid, propionic, and butyrate composed the major microbiota metabolites in the vagina (44, 45) and play different roles under healthy or disease states. Delgado-Diaz et al*.* (43) reported that acetate is capable of eliciting inflammatory effect in bacterial vaginosis, which can be encountered by lactic acid produced largely by *Lactobacillus*, (18) except *L. iners*. Interestingly, Wen et al*.* (46) found that a blocked conversion of acetate to butyrate may induce colon epithelial barrier damage in a pig model. In gynecological oncology models, it is recorded that butyrate can inhibit HPV-positive cervical cancer and ovarian cancer by arresting cell cycles (47, 48). Although researches showed that the abundance of *Lactobacillus* decreases with an increased level of acetate, butyrate, and other SCFAs in vaginal dysbiosis, the species of *L. iners* is an exception, which may induce inflammation and dysbiosis in the vagina, and may be associated with poor outcomes of bacterial vaginosis (49, 50). *In vitro* study showed *L. iners* is cysteine-dependent and can be targeted by inhibition agents (51). This study found that *L. iners* dominated in the H group; therefore, insufficient lactate could be produced to encounter the inflammation caused by SCFAs. This hypothesis might throw light on developing targeted treatment options but needs more solid work to verify it.

Limitations inevitably existed in this study. First, this is an observational study that depicted the vaginal microbiota features in different HPV infections, which demands more reliable proof to explain the phenomenon observed. Second, due to the limitation of 16s rDNA sequencing technique, the annotation to detailed species of microbiota needs a more exact method to verify it, such as culture or metagenomics sequencing. Lastly, the sampling method may cause potential selection biases in this study.

### Conclusions

Overall, this study investigated the prevalence of HPV genotypes in south China and for the first time identified vaginal microbiome features for hr-, lr-, and multiple HPV infections comprehensively. Each HPV group had distinct microbiome features. In particular, hr-HPV was characterized by *L. iners*, while lr-HPV was featured by *L. crispatus.* The imbalance of *Lactobacillus* spp., caused by HPV infection, might be associated with cervical lesions. These results might throw light on the mechanisms of genital HPV infection and pave the way for novel therapy and vaccination development.

## Data Availability

Raw sequence data can be obtained from NCBI (https://www.ncbi.nlm.nih.gov/) at SRA accession number PRJNA1089804. Other data that supports the findings of this study is available from the corresponding author upon reasonable request.

## SUPPLEMENTAL MATERIAL

Figure S1 msystems.00738-24-s0001.docx Microbiological diversity differences.

ASM does not own the copyrights to Supplemental Material that may be linked to, or accessed through, an article. The authors have granted ASM a non-exclusive, world-wide license to publish the Supplemental Material files. Please contact the corresponding author directly for reuse.
