# Supplementary material for: Human papillomavirus molecular prevalence in south China and the impact on vaginal microbiome of unvaccinated women
Source: mSystems. 2024 Aug 9;9(9):e00738-24. doi: 10.1128/msystems.00738-24 (PMC11407003; doi:10.1128/msystems.00738-24)
Supplement: Figure S1 — Microbiological diversity differences. [file msystems.00738-24-s0001.docx]

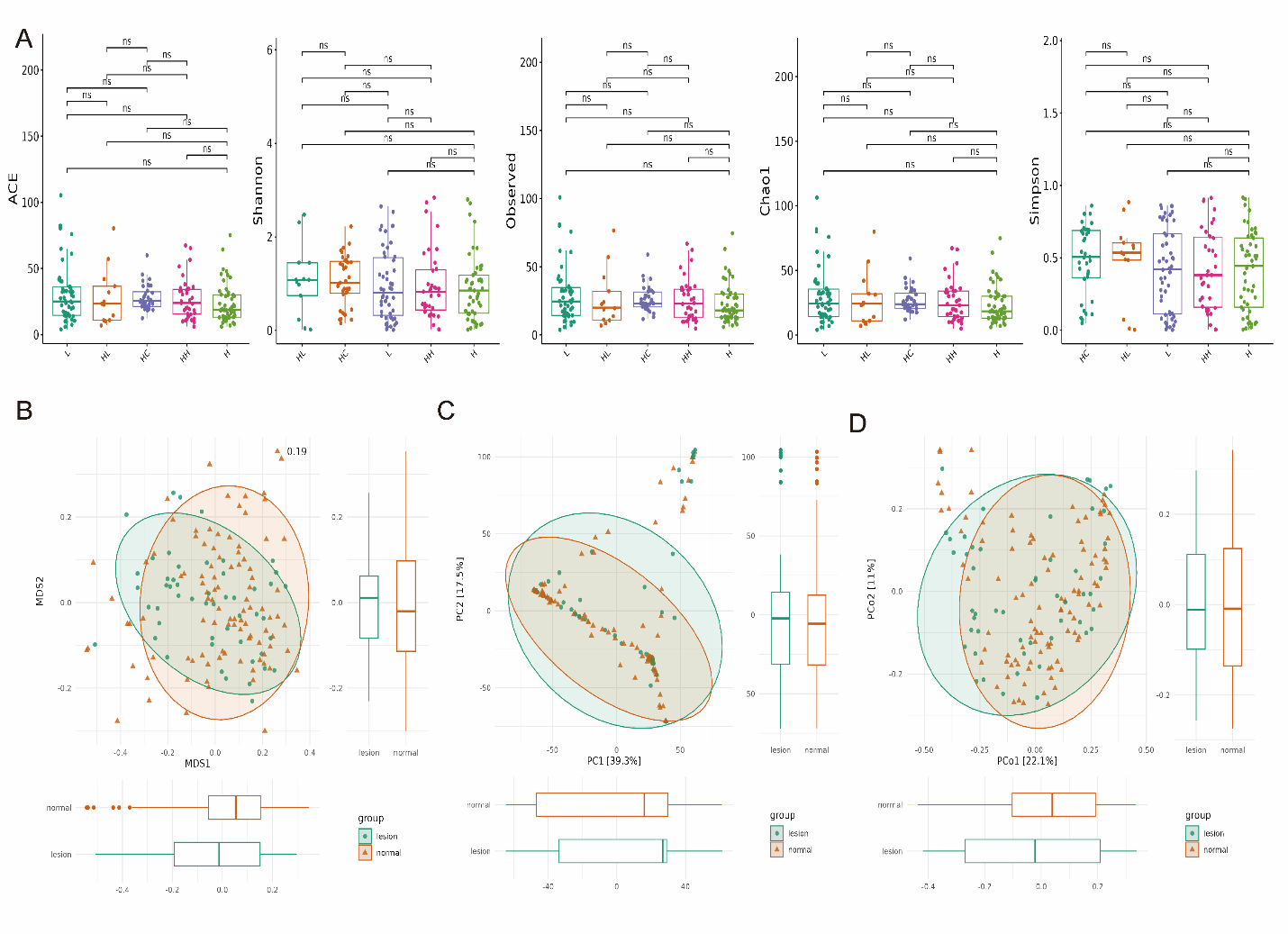


Supplementary figure 1. A. Differences in α diversity indexes among HC, H, HH, HL, and L groups. Differences in β diversity between HPV positive patients with cervical lesion and those with normal cervix by unweighted unifrac NMDS (B), unweighted unifrac PCA (C), and unweighted unifrac PCoA (D) analysis.
